# Dietary Glycemic Index during Pregnancy Is Associated with Biomarkers of the Metabolic Syndrome in Offspring at Age 20 Years

**DOI:** 10.1371/journal.pone.0064887

**Published:** 2013-05-31

**Authors:** Inge Danielsen, Charlotta Granström, Thorhallur Haldorsson, Dorte Rytter, Bodil Hammer Bech, Tine Brink Henriksen, Allan Arthur Vaag, Sjurdur Frodi Olsen

**Affiliations:** 1 Centre for Fetal Programming, Department of Epidemiology Research, Statens Serum Institute, Copenhagen, Denmark; 2 The Unit for Nutrition Research, Faculty of Food Science and Nutrition, School of Health Sciences, University of Iceland, Reykjavik, Iceland; 3 Centre for Fetal Programming, Department of Public Health, Section for Epidemiology, Aarhus University, Aarhus, Denmark; 4 Department of Paediatrics, Aarhus University Hospital, Skejby, Denmark; 5 Department of Endocrinology, Rigshospitalet, Copenhagen, Denmark; 6 Department of Clinical Medicine, University of Copenhagen, Copenhagen, Denmark; University of Arkansas for Medical Sciences, United States of America

## Abstract

**Objective:**

Growing evidence indicates that metabolic syndrome is rooted in fetal life with a potential key role of nutrition during pregnancy. The objective of the study was to assess the possible associations between the dietary glycemic index (GI) and glycemic load (GL) during pregnancy and biomarkers of the metabolic syndrome in young adult offspring.

**Methods:**

Dietary GI and GL were assessed by questionnaires and interviews in gestation week 30 and offspring were clinically examined at the age of 20 years. Analyses based on 428 mother-offspring dyads were adjusted for maternal smoking during pregnancy, height, pre-pregnancy body mass index (BMI), education, energy intake, and the offspring’s ambient level of physical activity. In addition, possible confounding by gestational diabetes mellitus was taken into account.

**Outcome Measures:**

Waist circumference, blood pressure, HOMA insulin resistance (HOMA-IR) and plasma levels of fasting glucose, triglycerides, HDL cholesterol, LDL cholesterol, total cholesterol, insulin, and leptin were measured in the offspring.

**Results:**

Significant associations were found between dietary GI in pregnancy and HOMA-IR (the relative increase in HOMA-IR per 10 units’ GI increase was 1.09 [95% CI: 1.01, 1.16], p = 0.02), insulin (1.09 [95% CI: 1.02, 1.16], p = 0.01) and leptin (1.21 [95% CI: 1.06, 1.38], p = 0.01) in the offspring; whereas no associations were detected for GL.

**Conclusions:**

Our data suggests that high dietary GI in pregnancy may affect levels of markers for the metabolic syndrome in young adult offspring in a potentially harmful direction.

## Introduction

Metabolic diseases, including the metabolic syndrome (MS), have been on the rise worldwide during the last two decades. MS is associated with increased risk of cardiovascular disease and type 2 diabetes and consists of the components: central obesity, reduced HDL cholesterol and raised triglyceride levels, as well as raised blood pressure and fasting plasma glucose levels [1]. Growing evidence indicates that MS is rooted in fetal life with a potential key role of nutrition during pregnancy [2]. The intrauterine environment, influenced by the maternal diet, may “programme” the fetus and thus influence susceptibility for MS in later life [3], [4].

The composition and amount of carbohydrates consumed during pregnancy are of particular interest. Dietary GI is a measure of the postprandial effect on the plasma glucose of the carbohydrate of a food item compared to the effect of the same amount of carbohydrate in pure glucose or white bread. GL in addition takes into account the amount of carbohydrate in the food item and thus is a more quantitative measure [5]. The concept of GI was first introduced by Jenkins et al. in 1981 [6] and is a commonly used measure of carbohydrate quality. Evidence has emerged from human and animal studies that maternal glucose levels may be predictive of metabolic disorders in the offspring [7]–[9]. Among a group of non-diabetic and generally healthy pregnant women, it has been shown that dietary GI is associated with the levels of glycosylated hemoglobin and plasma glucose [10]. There is also some evidence to support that a low GI diet during pregnancy may improve fetal glucose and insulin regulation and reduce birth weight and fetal adiposity [2], [10]–[13], although not all studies have supported this [14]. We are unaware of any previous studies in humans investigating the association between dietary GI or GL during pregnancy and the metabolic profile in the adult offspring. Accordingly, this association was studied in a unique prospective birth cohort with 20 years’ follow-up.

## Materials and Methods

### Ethics Statement

The study was approved by the Danish Data Protection Agency and the Central Denmark Region Committees on Biomedical Research Ethics (Reference No. 20070157). Written consent was obtained from all participants.

### The Danish Fetal Origin Cohort 1988

In 1988 a total of 965 out of 1212 eligible women with singleton pregnancies were recruited for a birth cohort study in Denmark [15]. Prior to the routine antenatal visit in gestational week 30, the pregnant women received a postal questionnaire to complete and return to the antenatal care clinic. Following the antenatal visit, a 15-minute face-to-face interview was conducted by a trained person who corroborated the response to the self-administered questionnaire and completed a second interviewer-guided questionnaire with the women. The two questionnaires covered medical history, diet and other lifestyle as well as socio-economic factors. Further information about the women’s health, birth outcomes, medical history, and anthropometry was extracted from hospital records and from the Danish Medical Birth Registry as well as from the records kept by the midwives and general practitioners. Moreover, screening for gestational diabetes mellitus (GDM) was done with fasting glucose measurements in woman who were obese, had a family history of diabetes mellitus, GDM in a previous pregnancy, a previous delivery of an infant above 4500 g, previous stillbirth, age above 38 years, or glucosuria in the current pregnancy. When two independent fasting capillary plasma glucose values were above 4.6 mmol/L the woman was referred to an OGTT [16].

### Offspring Follow Up

During 2008 and 2009, mothers and offspring were contacted and offspring invited to complete a web-based questionnaire including inquiries on current health, lifestyle and dietary habits as well as height, weight and waist circumference.

All potential participants were asked to participate in a clinical examination. The participants were examined between 8∶00 AM and 12∶30 PM after an overnight fasting. Height, weight and waist circumference were measured. After 7 min. of rest, blood pressure was measured three times in the horizontal position (2 min. intervals in between) using an automatic blood pressure device (OMRON M6 Comfort HEM-7000-E). The average value of the last two measurements was used in the analyses. A venous blood sample was drawn and immediately centrifuged and frozen at −80°C.

From a total number of 965 women we traced 894 singleton offspring. The remaining study group included twins, mothers and children with an incorrect personal identification number (in use for every citizen in Denmark), stillbirths, mothers and children who had died or were abroad, or with unknown addresses, or offspring that was unable to participate because of illness.

A total of 688 subjects (77% of the eligible population) participated in the follow up study by filling out the questionnaire, providing information on the offspring’s level of physical activity, and of these 439 attended the clinical examination.

### Offspring Biomarkers

Plasma glucose levels were measured using bedside equipment (Accu-chek, Roche Diagnostics, Germany) immediately after blood sampling. Serum leptin concentrations were determined at the Medical Research Laboratories, Aarhus University Hospital, Denmark, by a time-resolved immunofluorometric assay based on commercially available reagents and recombinant human leptin as standard [17]. Plasma insulin concentrations were determined using a commercial ELISA kit. Insulin resistance was estimated using the homeostasis model assessment for insulin resistance (HOMA-IR) by means of the formula: fasting glucose (mmol/L) x fasting insulin (mU/L)/22.5 [18]. Serum triglycerides and cholesterol fractions (total cholesterol, LDL, HDL) were measured according to standard methods on a Modular P from Roche Diagnostics, Basel, Switzerland.

### Markers for MS

Primary outcome variables were chosen among the continuous variables inherent in the definition of MS, i.e. waist circumference (cm), fasting levels of plasma glucose (mmol/L), triglycerides (mmol/L) and HDL cholesterol (mmol/L), as well as systolic and diastolic blood pressure measurements (mmHg). Additionally, the primary outcome variables were supplemented with secondary variables associated with MS including BMI (kg/m^2^), plasma levels of LDL and total cholesterol (mmol/L), concentrations of fasting plasma insulin (pmol/L) and leptin (ug/L), as well as HOMA-IR. Waist circumference and BMI were determined solely by means of data from the clinical examinations (439 subjects) to eliminate the possibility of under-reported data on BMI and waist circumference. BMI>18.5 and <25 kg/m^2^ was considered normal, whereas BMI≤18.5, BMI≥25.0 kg/m^2^ and BMI≥30.0 kg/m^2^ is termed underweight, overweight and obese, respectively.

### Exposure Variables

The dietary assessment method used was a self-administered semi-quantitative food questionnaire combined with an interview in which photographic aids were used to assess portion sizes. The women were systematically asked about all possible categories of food items. The questions gave information on how often per week or per day the food item was consumed and how much per portion. Assessment of dietary intake was done by means of a national food composition database using standard recipes and standard portion sizes supplementing the answers from the questionnaire.

Dietary GI is a measure of the increase in plasma glucose after intake of a food item (containing 50 or 100 g of carbohydrate) and defined as the incremental area under the postprandial glucose response curve in percentage of the corresponding area following intake of a standard reference food item (same amount of carbohydrate), which can be either glucose or white bread [5]. Thus, GI measures the effect of the carbohydrate in the specific food item on the plasma glucose and thus represents a quality aspect of the food. GL represents both the quality and the quantity of the food, taking into account that the glycemic effect of a food item depends not only on the GI but also on the amount of carbohydrate eaten. Application of GI and GL in research and partly in clinical settings is widespread though the concept of GI is still contentious because of controversies relating to methodology and clinical applicability [19], [20].

For foods included in the questionnaire, we used the GI-table from Foster-Powell et al. 2002 [5], though a newer table exists [21], with white bread as the standard reference. The table contains GI-values measured during a time period close to the time when the dietary data were collected in the cohort. This is important as the composition of processed foods, and thereby the GI of these food items, changes over time. Daily dietary GI was calculated as the product of the GI and carbohydrate content for each food or beverage, summed for all items consumed either as a snack or as part of a meal in average per day and divided by the total carbohydrate intake per day. Daily GL was calculated as the product of the GI and carbohydrate content for each food or beverage and summed for all items consumed in average per day and then energy-adjusted by the residual method [22]. Both variables were analysed as continuous variables and in quintiles based on the diet of all women in the cohort (n = 894).

### Statistical Analyses

Baseline characteristics of pregnant women either with participating or non-participating offspring were tested for differences by χ^2^-test. Distributions of covariates according to maternal exposure of GI and GL were tested for trends across quintiles of GI and GL by using Mantel-Haentzel χ^2^-test for trend for categorical covariates. Associations between maternal GI and GL and offspring outcome variables were examined by multivariate linear regression analyses. Waist circumference was adjusted for BMI using the residual method [23], providing an uncorrelated measure of BMI and waist circumference. Due to skewed distributions, all outcome variables except adjusted waist circumference and blood pressure were log-transformed. We *a priori* decided to include the following covariates: maternal height (continuous, 3% missing), education (five categories, 5% missing), smoking (yes or no, 5% missing), pre-pregnancy BMI (continuous, 3% missing), energy intake (five quintiles, 0% missing), and offspring’s current physical activity (four categories, 0% missing). Observations with missing covariate values were excluded from the analyses.

Maternal height, pre-pregnancy BMI and offspring’s physical activity were included as these variables are possible determinants of anthropometric and metabolic measures in the offspring. Energy intake was included as it is associated with the diet and possibly also the outcome variables. Maternal education and smoking were included to account for potential social and lifestyle confounding. In the presented data, women diagnosed with GDM were excluded from the analyses. Furthermore, the influence of GDM status was investigated by conducting the analyses after inclusion of the GDM cases.

The analyses were performed for combined sexes as well as males and females separately. The combined analysis included sex in the model along with the other covariates. Among the women with participating offspring, the GI varied between 49.3 and 88.3, whereas GL varied between 108.6 and 267.6. Considering this exposure range, analyses were made using exposures of 10 units’ increments with the aim to create results with magnitudes of clinical relevance. Mean changes in outcome variables for 10 units’ increment in GI and GL are presented as absolute increments for waist circumference and blood pressure and relative increments for BMI and offspring biomarkers. These measures of association are expressed as ‘difference per 10U GI/GL increase’ and ‘ratio per 10U GI/GL increase’. Associations were considered statistically significant at the 5% level and all regression coefficients are presented with 95% CI. All analyses were performed using the SAS GLM procedure (Version 9.3; SAS Institute, Cary, NC).

## Results

Mothers of participating offspring were more often normal weight and non-smokers and had a higher education compared to mothers of non-participating offspring (table 1). Furthermore they had a higher energy intake. No significant differences were observed between the two groups regarding dietary GI and GL.

**Table 1:** Characteristics of 894 pregnant women in the birth cohort dependent on their offspring’s participation in the follow up.

	Mothers with non-participating offspring (n = 206)	Mothers with participating offspring (n = 688)	
	Percent	Percent	p-value
Height			0.73
−159	9	8	
160–164	24	20	
165–169	33	34	
170–174	23	26	
175-	11	13	
Education			<0.0001
None	22	13	
Vocational	36	23	
Bachelor	35	44	
Academic	6	19	
BMI (kg/m^2^)			<0.01
<18.6	15	9	
18.6–<25	72	82	
25–<30	8	6	
30–	5	2	
Smoking in pregnancy	50	37	<0.001
Nulliparous	56	59	0.46
Energy intake			0.03
Lowest quintile	24	19	
Mid quintile	15	22	
Highest quintile	17	21	
Glycemic Index			0.98
Lowest quintile	20	20	
Mid quintile	21	20	
Highest quintile	20	20	
Glycemic Load			0.07
Lowest quintile	24	19	
Mid quintile	21	20	
Highest quintile	23	19	

Differences between the two groups of women are reported as p-value from χ^2^-test for measure of association.

There was a significant association between increasing GI and GL and decreasing education, and fewer women with a low GL intake were smoking compared to women with at high GL intake (supplementary table S1). No significant association was found with any other covariate included in the model.

Of the 439 women included in the analyses, 121 women with high risk of GDM were screened and 11 (9.0% of the screened group and 2.5% of the total group) were diagnosed. The estimated prevalence of GDM in Denmark in 1999–2000 was 2.4% [24], corresponding to between 10 and 11 GDM cases in a population of 439 women. The 11 GDM cases were excluded from the analyses reported in tables 2–4. For unadjusted results, please see the supplementary tables S2, S3, and S4.

**Table 2:** MS markers in male and female offspring dependent on their mothers’ dietary GI in 2nd trimester.

		Ratio or difference* (95% CI)						
		GI quintile 1	GI quintile 2	GI quintile 3	GI quintile 4	GI quintile 5	GI continuous	p-value
	Mean ± SD	60.8+3.6	67.4+1.3	71.1+1.0	74.5+1.1	80.0+2.7	70.5±6.9	
Fasting glucose	4.9 mmol/L (±1.1)	1	0.99 (0.97, 1.01)	1.01 (0.99, 1.04)	1.03 (1.00, 1.05)	1.00 (0.98, 1.03)	1.01 (1.00, 1.02)	0.18
Triglycerides	0.9 mmol/L (±1.5)	1	1.04 (0.91, 1.18)	1.10 (0.97, 1.24)	1.16 (1.02, 1.32)	1.04 (0.90, 1.19)	1.04 (0.98, 1.11)	0.18
HDL cholesterol	1.4 mmol/L (±1.2)	1	1.05 (0.98, 1.12)	1.04 (0.98, 1.11)	1.05 (0.98, 1.12)	1.03 (0.96, 1.11)	1.03 (1.00, 1.06)	0.09
LDL cholesterol	2.4 mmol/L (±1.3)	1	1.05 (0.96, 1.15)	1.07 (0.98, 1.16)	1.10 (1.01, 1.21)	1.07 (0.97, 1.18)	1.03 (0.98, 1.08)	0.26
Total cholesterol	4.3 mmol/L (±1.2)	1	1.04 (0.98, 1.10)	1.06 (1.00, 1.12)	1.09 (1.03, 1.15)	1.05 (0.99, 1.12)	1.03 (1.00, 1.06)	0.05
Systolic bloodpressure*	109.9 mmHg (±10.6)	0	−2.58(−5.27, 0.10)	−1.31(−3.93, 1.30)	−0.10(−2.84, 2.63)	−2.11(−5.02, 0.81)	−0.55(−1.91, 0.82)	0.43
Diastolic bloodpressure*	65.7 mmHg (±6.7)	0	−0.21(−2.25, 1.83)	0.33(−1.66, 2.31)	1.15(−0.92, 3.22)	−0.25(−2.46, 1.95)	0.29(−0.74, 1.32)	0.59
Waist circumference*	81.6 cm (±6.0)	0	0.28(−1.08, 1.65)	0.32(−1.01, 1.64)	0.71(−0.68, 2.10)	1.14(−0.34, 2.62)	0.47(−0.22, 1.16)	0.18
BMI	22.2 kg/m^2^ (±1.1)	1	0.99 (0.95, 1.03)	1.02 (0.98, 1.06)	1.03 (0.99, 1.08)	1.01 (0.97, 1.05)	1.01 (0.99, 1.03)	0.28
HOMA-IR	1.2 (±1.6)	1	1.04 (0.91, 1.19)	1.16 (1.01, 1.32)	1.27 (1.11, 1.46)	1.11 (0.96, 1.28)	1.09 (1.01, 1.16)	0.02
Insulin	39.5 pmol/L (±1.5)	1	1.08 (0.94, 1.23)	1.15 (1.01, 1.30)	1.26 (1.11, 1.44)	1.12 (0.97, 1.29)	1.09 (1.02, 1.16)	0.01
Leptin	6.7 ug/L (±3.3)	1	0.93 (0.72, 1.21)	1.22 (0.95, 1.57)	1.38 (1.06, 1.79)	1.21 (0.91, 1.60)	1.21 (1.06, 1.38)	0.01

Shown are the mean differences in the outcome variables waist circumference and systolic and diastolic blood pressure (indicated by*) and mean ratio for all other log transformed outcome variables. The table includes figures from analyses of quintiles of GI, and from analyses of the data using GI as continuous variable (ratio or difference per 10U GI increment)^1^.

1 Adjustment for potential confounding by multiple linear regression including energy intake, pre-pregnancy BMI (kg/m^2^), height (cm), smoking, education, and offspring sex and leisure activity. The p-value is the result of analyses of the data using GI as continuous variable (n = 386). GI quintiles were determined from the original data files including 894 women.

**Table 3:** MS markers in female offspring dependent on their mothers’ dietary GI in 2nd trimester.

		Ratio or difference* (95% CI)						
		GI quintile 1	GI quintile 2	GI quintile 3	GI quintile 4	GI quintile 5	GI continuous	p-value
	Mean ± SD	60.8+3.6	67.4+1.3	71.1+1.0	74.5+1.1	80.0+2.7	70.4+6.7	
Fasting glucose	4.8 mmol/L (±1.1)	1	0.98 (0.95, 1.02)	1.01 (0.98, 1.05)	1.02 (0.99, 1.06)	0.99 (0.96, 1.03)	1.00 (0.99, 1.02)	0.76
Triglycerides	1.0 mmol/L (±1.5)	1	1.07 (0.91, 1.26)	1.08 (0.92, 1.28)	1.21 (1.01, 1.45)	1.02 (0.85, 1.22)	1.04 (0.95, 1.13)	0.37
HDL cholesterol	1.5 mmol/L (±1.2)	1	1.05 (0.97, 1.14)	1.02 (0.94, 1.11)	1.07 (0.98, 1.17)	1.05 (0.96, 1.15)	1.04 (1.00, 1.09)	0.06
LDL cholesterol	2.4 mmol/L (±1.4)	1	1.08 (0.96, 1.21)	1.06 (0.94, 1.20)	1.14 (1.00, 1.29)	1.15 (1.01, 1.31)	1.05 (0.99, 1.12)	0.10
Total cholesterol	4.5 mmol/L (±1.2)	1	1.06 (0.99, 1.15)	1.05 (0.97, 1.13)	1.12 (1.03, 1.22)	1.11 (1.02, 1.20)	1.05 (1.01, 1.09)	0.01
Systolic blood pressure*	104.8 mmHg (±8.1)	0	−2.16(−5.44, 1.11)	−1.86(−5.13, 1.42)	−0.34(−3.94, 3.25)	−1.50(−5.11, 2.11)	−0.51(−2.23, 1.21)	0.56
Diastolic blood pressure*	66.5 mmHg (±6.4)	0	0.58(−1.93, 3.09)	0.11(−2.40, 2.62)	2.07(−0.68, 4.82)	0.98(−1.79, 3.74)	0.52(−0.80, 1.84)	0.44
Waistcircumference*	79.4 cm (±5.3)	0	1.03(−0.78, 2.85)	1.04(−0.78, 2.86)	1.18(−0.82, 3.17)	2.00(−0.01, 4.00)	0.78(−0.17, 1.74)	0.11
BMI	21.9 kg/m^2^ (±1.2)	1	1.00 (0.95, 1.05)	1.02 (0.96, 1.07)	1.04 (0.98, 1.10)	1.02 (0.96, 1.08)	1.01 (0.98, 1.04)	0.52
HOMA-IR	1.2 (±1.6)	1	1.14 (0.95, 1.36)	1.23 (1.03, 1.46)	1.33 (1.10, 1.61)	1.13 (0.93, 1.37)	1.08 (0.99, 1.19)	0.10
Insulin	41.1 mmol/L (±1.5)	1	1.21 (1.03, 1.43)	1.22 (1.03, 1.44)	1.29 (1.08, 1.55)	1.17 (0.97, 1.40)	1.08 (0.99, 1.19)	0.07
Leptin	13.4 ug/L (±2.1)	1	1.04 (0.79, 1.38)	1.19 (0.90, 1.58)	1.42 (1.05, 1.94)	1.40 (1.03, 1.91)	1.21 (1.05, 1.40)	0.01

Shown are the mean differences in the outcome variables waist circumference and systolic and diastolic blood pressure (indicated by*) and mean ratio for all other log transformed outcome variables. The table includes figures from analyses of quintiles of GI, and from analyses of the data using GI as continuous variable (ratio or difference per 10U GI increment)^1^.

1 Adjustment for potential confounding by multiple linear regression including energy intake, pre-pregnancy BMI (kg/m^2^), height (cm), smoking, education, and offspring leisure activity. The p-value is the result of analyses of the data using GI as continuous variable (n = 234). GI quintiles were determined from the original data files including 894 women.

**Table 4:** MS markers in male offspring dependent on their mothers’ dietary GI in 2nd trimester.

		Ratio or difference* (95% CI)						
		GI quintile 1	Gi quintile 2	GI quintile 3	Gi quintile 4	GI quintile 5	GI continuous	p-value
	Mean ± SD	60.8+3.6	67.4+1.3	71.1+1.0	74.5+1.1	80.0+2.7	70.6±6.4	
Fasting glucose	5.1 mmol/L (±1.1)	1	1.00 (0.96, 1.04)	1.02 (0.98, 1.06)	1.03 (0.99, 1.07)	1.03 (0.98, 1.07)	1.02 (1.00, 1.04)	0.06
Triglycerides	0.8 mmol/L (±1.5)	1	0.99 (0.80, 1.21)	1.14 (0.94, 1.38)	1.11 (0.92, 1.35)	1.09 (0.87, 1.35)	1.06 (0.96, 1.17)	0.26
HDL cholesterol	1.3 mmol/L (±1.2)	1	1.04 (0.93, 1.17)	1.07 (0.96, 1.19)	1.02 (0.92, 1.14)	0.99 (0.88, 1.12)	1.01 (0.95, 1.06)	0.82
LDL cholesterol	2.3 mmol/L (±1.3)	1	1.02 (0.88, 1.18)	1.08 (0.94, 1.24)	1.06 (0.93, 1.21)	0.96 (0.83, 1.12)	0.99 (0.93, 1.07)	0.87
Total cholesterol	4.0 mmol/L (±1.2)	1	1.01 (0.92, 1.11)	1.08 (0.99, 1.17)	1.04 (0.96, 1.14)	0.97 (0.88, 1.07)	1.00 (0.95, 1.04)	0.96
Systolic bloodpressure*	117.5 mmHg (±9.3)	0	−3.59(−8.52, 1.33)	−1.92(−6.50, 2.66)	−1.01(−5.63, 3.61)	−4.00(−9.23, 1.23)	−1.14(−3.50, 1.21)	0.34
Diastolic bloodpressure*	64.5 mmHg (±7.0)	0	−1.28(−4.87, 2.30)	−0.13(−3.47, 3.21)	−0.94(−4.31, 2.43)	−2.26(−6.08, 1.55)	−0.21(−1.92, 1.51)	0.81
Waistcircumference*	84.0 cm (±5.7)	0	−1.55(−3.73, 0.63)	−1.20(−3.22, 0.83)	−0.52(−2.56, 1.52)	−0.81(−3.12, 1.50)	−0.19(−1.23, 0.85)	0.72
BMI	22.8 kg/m^2^ (±2.9)	1	0.98 (0.92, 1.04)	1.02 (0.96, 1.08)	1.02 (0.96, 1.08)	1.00 (0.93, 1.07)	1.02 (0.99, 1.05)	0.32
HOMA-IR	1.2 (±1.6)	1	0.89 (0.71, 1.11)	1.06 (0.86, 1.30)	1.23 (1.00, 1.52)	1.05 (0.83, 1.32)	1.09 (0.98, 1.22)	0.10
Insulin	37.2 pmol/L (±1.5)	1	0.88 (0.71, 1.10)	1.04 (0.85, 1.27)	1.24 (1.01, 1.52)	1.01 (0.80, 1.27)	1.08 (0.97, 1.20)	0.14
Leptin	2.3 ug/L (±2.7)	1	0.78 (0.46, 1.31)	1.20 (0.73, 1.96)	1.20 (0.74, 1.97)	0.91 (0.52, 1.59)	1.14 (0.88, 1.46)	0.31

Shown are the mean differences in the outcome variables waist circumference and systolic and diastolic blood pressure (indicated by*) and mean ratio for all other log transformed outcome variables. The table includes figures from analyses of quintiles of GI, and from analyses of the data using GI as continuous variable (ratio or difference per 10U GI increment)^1^.

1 Adjustment for potential confounding by multiple linear regression including energy intake, pre-pregnancy BMI (kg/m^2^), height (cm), smoking, education, and offspring leisure activity. The p-value is the result of analyses of the data using GI as continuous variable (n = 152). GI quintiles were determined from the original data files including 894 women.

In the adjusted analyses of offspring of both sexes (table 2), we found no associations among the primary outcome variables. Among the secondary variables, with increasing GI we found higher levels of total cholesterol (ratio per 10U GI increase 1.03 (95% CI: 1.00, 1.06)), higher HOMA-IR (ratio 1.09 (95% CI: 1.01, 1.16)), and higher levels of insulin (ratio 1.09 (95% CI: 1.02, 1.16)) and leptin (ratio 1.21 (95% CI: 1.06, 1.38)). Analyses with quintiles of GI did not consistently result in significant associations but were generally in agreement with the analyses with the continuous variable. No associations were observed for GL (data not shown).

In female offspring exposed to maternal GI (table 3) we found no associations among the primary outcome variables. Among the secondary variables, with increasing GI we found higher levels of total cholesterol (ratio 1.05 (95% CI: 1.01, 1.09)) and leptin (ratio 1.21 (95% CI: 1.05, 1.40)) and in addition a borderline significant association with higher levels of insulin (ratio 1.08 (95% CI: 0.99, 1.19)). Furthermore, with increasing GL the data showed significant associations with the primary variables: systolic blood pressure (difference per 10U GL increase −0.46 (95% CI: −0.91, −0.01)) and waist circumference (difference 0.26 (95% CI: 0.01, 0.51).

No significant associations between maternal GI and GL and any outcome measure were observed in male offspring (table 4 and data not shown).

Inclusion of the 11 GDM cases in the analyses did not change the associations (data not shown). Adding the offspring’s waist circumference to the statistical model did not the change the associations either (data not shown).

## Discussion

In this study we showed significant associations between high dietary GI in pregnancy and key markers of the metabolic syndrome (MS) in the young adult offspring. The results may be of clinical relevance. Thus, an increase in maternal GI of 10 units was associated with increased HOMA-IR, as well as increased levels of insulin and leptin among offspring of 9, 9 and 21%, respectively.

GL was not associated with MS markers among the offspring in the combined analyses. Nevertheless, among the females the observed association between increasing GL and higher waist circumference could be clinically relevant, as the possible effect of an increase of only 10 GL units – out of a total exposure range of 159 GL units - was 0.3 cm. A borderline significant association between increasing GL and lower systolic blood pressure among female offspring only. The extent to which these associations may be of clinical relevance remains to be determined.

Additional adjustment for the offspring’s waist circumference did not change the associations between GI in pregnancy and MS markers in the offspring, suggesting that factors other than current abdominal adiposity and body composition may explain the associations. These results are in full agreement with a recent study reporting that carbohydrate-rich diet supplementation in pregnant rats was associated with increased insulin, leptin and glucose levels among their adult male offspring, while no association was found with the offspring’s body weight [25]. These results in rats together with ours in humans support the idea that GI (and perhaps GL) during pregnancy may exhibit a causal programming effect in the adult offspring. Nevertheless, in our observational study we cannot exclude the theoretical possibility that mother’s and child’s lifestyles, including eating and physical activity habits, even after 20 years, may be similar as a result of social and familial factors. However, excluding the offspring’s physical activity from the statistical model did not change the associations between maternal GI and offspring MS markers (data not shown). The extent to which the mother’s diet during pregnancy is associated with the diet and eating habits of the offspring is unknown. Nevertheless, leptin in the offspring, the level of which was associated with the mother’s GI, may influence upon appetite and eating patterns [26]. Accordingly, it is possible that the offspring’s diet and levels of leptin may act in concert as mediators in the pathways between maternal GI and offspring MS markers. In this perspective it would be incorrect to adjust for the offspring’s diet.

Epigenetic mechanisms may be involved in developmental programming of MS by GI in pregnancy. Thus, a recent study found associations between maternal carbohydrate content in early pregnancy and DNA methylation of candidate genes from umbilical cord tissue and between the methylation status at birth and child’s obesity at the age of 9 [27].

According to the “Pedersen hypothesis” and the theory of “fuel mediated teratogenesis”, maternal glucose crosses the placenta and results in intrauterine hyperglycemia and fetal hyperinsulinemia leading to increased fetal growth and adiposity with consequences for later health [28]–[30]. This may explain the right side of the U-shaped association between birth weight and subsequent development of type 2 diabetes [31], [32]. Interestingly, the HAPO study suggested that mild elevations of maternal plasma glucose levels may impact on birth weight as well as risk of maternal and fetal pregnancy complications even at a level below conventional diagnostic GDM plasma glucose cut off levels [33]. In the present study, also after exclusion of the 11 GDM cases, the data revealed associations between dietary GI in pregnancy and distinct offspring MS markers. Given the known influence of GI on plasma glucose levels even in non-diabetic pregnant women (10), we speculate that subtle elevations of plasma glucose in pregnant women may be a mediator of the programming effect of GI in pregnancy.

The associations observed between maternal GI and offspring levels of HOMA-IR, leptin and insulin did not differ significantly between male and female offspring (p>0.05). Notably, however, when restricting the analyses to either sex, the associations for leptin and insulin persisted in females but not in males. This finding may reflect a lower participation rate among the latter or sex-specific mechanisms in the fetal programming of metabolic traits. Recently, in the same birth cohort, we detected obesogenic effects in female but not in male offspring as a result of fetal exposure to perfluorooctanoate [34]. Our findings are in line with a recent study on data from the great Chinese famine, where exposure to severe undernutrition in fetal life was associated with higher risk of MS in adult women but not in men [35] and with another study on data from the Dutch famine study, where maternal malnutrition during early gestation was associated with obesity only in women [36]. Sexual dimorphism in developmental programming may be explained by the sexually dimorphic embryo-derived tissue of the placenta which plays a significant role in determing fetal size, nutrition, morbidity and survival [37]. During recent years, evidence has emerged from rodent and human studies that the growth of the placenta and gene expression as well as DNA methylation differ between the sexes and furthermore that the sexes respond differently to environmental insults [37]–[39]. Differences in response to quality or composition of the maternal diet on the sex-dependent placental gene expression have been observed as well, providing insight in different sensitivity of male and female fetuses to the maternal diet. Thus the female placenta seems to be more adaptive to changes in maternal diet compared to the male placenta [40]. Furthermore, it has been shown that the maternal diet composition influences the cortisol level in the mother [41]–[43] and, in addition, changes the gene expression and the activity of the HPA axis, which regulate the glucocorticoid production, differently in male and female fetuses and offspring [37], [44]. Among humans, higher maternal cortisol was associated with marginally higher Fat Mass Index (FMI) in 5 year old girls, but marginally lower FMI in boys [45], indicating that diet-dependent maternal cortisol levels may influence the offspring with long-term consequences.

Our data indicates that GI during pregnancy has stronger influence on the offspring’s metabolic profile than GL. In previous studies investigating the associations between dietary GI and GL on biomarkers for metabolic disease in adults, the associations were not consistent between GI and GL [46]–[48]. Recent meta-analyses have reported statistically significant associations between GI or GL and chronic diseases including cardiovascular disease and type 2 diabetes and came to different conclusions when addressing the question of whether GI or GL had the most powerful effect on development of disease [47], [49]. Interestingly, the associations between GI and GL on one hand and adult disease on the other in general have been reported to be stronger in women than in men [46], [47], which may further support our sex-dependent findings in the offspring.

A limitation of the study is the use of biomarkers for MS and not MS per se in the offspring, as none of the young people had developed overt MS at the time of follow-up. The study had several strengths. Information on maternal diet was provided prospectively with no knowledge about offspring conditions, physical activity or body proportions, and the study had a long follow-up period. We could not identify any participation bias with regards to the exposure levels (cf. table 1).

In conclusion, our data suggests that dietary GI in second trimester of pregnancy may be a determinant of HOMA-IR and plasma levels of insulin, leptin and cholesterol in the adult offspring. GI and GL did not seem to predict markers of MS in males, whereas both GI and GL were associated with specific markers of MS among the females. We speculate that the programming effect(s) of maternal dietary GI in pregnancy may be mediated via subtle elevations of plasma glucose levels within the non-diabetic range. This remains to be addressed in future studies.

## Supporting Information

Table S1 Characteristics of the pregnant women in relation to dietary GI and GL in 2nd trimester.
(DOCX)

Table S2 MS markers in male and female offspring dependent on their mothers’ dietary GI in 2nd trimester.
(DOCX)

Table S3 MS markers in female offspring dependent on their mothers’ dietary GI in 2nd trimester.
(DOCX)

Table S4 MS markers in male offspring dependent on their mothers’ dietary GI in 2nd trimester.
(DOCX)
