# Correction: Dietary Glycemic Index during Pregnancy Is Associated with Biomarkers of the Metabolic Syndrome in Offspring at Age 20 Years

**DOI:** 10.1371/annotation/e72c7dcd-f5f9-4deb-a185-f468b04d336a

**Published:** 2013-07-02

**Authors:** Inge Danielsen, Charlotta Granström, Thorhallur Haldorsson, Dorte Rytter, Bodil Hammer Bech, Tine Brink Henriksen, Allan Arthur Vaag, Sjurdur Frodi Olsen

The name of the third author was incorrect. The correct name is: Thorhallur I Halldorsson.

The correct citation is: Danielsen I, Granström C, Halldorsson TI, Rytter D, Hammer Bech B, et al. (2013) Dietary Glycemic Index during Pregnancy Is Associated with Biomarkers of the Metabolic Syndrome in Offspring at Age 20 Years. PLoS ONE 8(5): e64887. doi:10.1371/journal.pone.0064887.

The correct abbreviation in Contributions is: TIH. 

