# Supplementary material for: Dietary Glycemic Index during Pregnancy Is Associated with Biomarkers of the Metabolic Syndrome in Offspring at Age 20 Years
Source: PLoS One. 2013 May 31;8(5):e64887. doi: 10.1371/journal.pone.0064887 (PMC3669023; doi:10.1371/journal.pone.0064887)
Supplement: Table S1 — Characteristics of the pregnant women in relation to dietary GI and GL in 2nd trimester. (DOCX) [file pone.0064887.s001.docx]

ONLINE SUPPLEMENTARY DATA

Contents:

Table S1: Characteristics of the pregnant women in relation to dietary GI and GL in 2nd trimester

Table S2: MS markers in male and female offspring dependent on their mothers’ dietary GI in 2nd trimester

Table S3: MS markers in female offspring dependent on their mothers’ dietary GI in 2nd trimester

Table S4: MS markers in male offspring dependent on their mothers’ dietary GI in 2nd trimester

Investigators:

Inge Danielsen, MS, MPA^1^, Charlotta Granström, MS^1^, Thorhallur Haldorsson, MS, PHD^1,2^, Dorte Rytter MS, PHD^1,3^, Bodil Hammer Bech, MD, PHD^3^, Tine Brink Henriksen, MD, PHD^4^, Allan Arthur Vaag, MD, PHD^5,6^, Sjurdur Frodi Olsen, MD, PHD^1^

^1^Centre for Fetal Programming, Department of Epidemiology Research, Statens Serum Institute, Copenhagen, Denmark.

^2^The Unit for Nutrition Research, Faculty of Food Science and Nutrition, School of Health Sciences, University of Iceland, Reykjavik, Iceland

^3^Department of Public Health, Section for Epidemiology, Aarhus University, Aarhus, Denmark

^4^Department of Paediatrics, Aarhus University Hospital, Skejby, Denmark

^5^Department of Endocrinology, Rigshospitalet, Copenhagen, Denmark

^6^Copenhagen University, Copenhagen, Denmark

Table S1. Characteristics of 386 pregnant women and their offspring included in the analyses in relation to the exposure variables, dietary GI ad GL shown in increasing quintiles with GI 1 as the lowest. Shown are the percentages of women in each category and quintiles of GI and GL, respectively.

|  |  | Distribution of women (%) across quintiles of Glycemic Index and Glycemic Load | | | | | | | | | | | |
| --- | --- | --- | --- | --- | --- | --- | --- | --- | --- | --- | --- | --- | --- |
|  |  | Glycemic Index | | | | | | Glycemic Load | | | | | |
| Covariate | Category | GI 1 | GI 2 | GI 3 | GI 4 | GI 5 | p-trend | GL 1 | GL 2 | GL 3 | GL 4 | GL 5 | p-trend |
| Height (cm) | -159 | 19.4 | 19.4 | 19.4 | 29.0 | 12.9 | 0.60 | 16.1 | 22.6 | 32.3 | 16.1 | 12.9 | 0.83 |
|  | 160-164 | 18.5 | 27.2 | 23.5 | 16.0 | 14.8 |  | 17.3 | 19.8 | 22.2 | 24.7 | 16.0 |  |
|  | 165-169 | 22.1 | 21.4 | 23.7 | 15.3 | 17.6 |  | 18.3 | 26.0 | 15.3 | 22.1 | 18.3 |  |
|  | 170-174 | 21.0 | 18.0 | 22.0 | 22.0 | 17.0 |  | 18.0 | 23.0 | 21.0 | 19.0 | 19.0 |  |
|  | 175- | 23.3 | 11.6 | 16.3 | 32.6 | 16.3 |  | 25.6 | 9.3 | 27.9 | 16.3 | 20.9 |  |
| Pre-pregnancy BMI (kg/m^2^) | 1-18.5 | 8.3 | 16.7 | 27.8 | 19.4 | 27.8 | 0.81 | 16.7 | 19.4 | 16.7 | 25.0 | 22.2 | 0.30 |
|  | 18.6-24.9 | 22.1 | 21.8 | 21.2 | 20.9 | 14.0 |  | 18.1 | 22.7 | 20.9 | 20.9 | 17.4 |  |
|  | 25-29.9 | 18.2 | 13.6 | 22.7 | 13.6 | 31.8 |  | 27.3 | 9.1 | 31.8 | 13.6 | 18.2 |  |
|  | 30- | 42.9 | 0 | 28.6 | 14.3 | 14.3 |  | 28.6 | 28.6 | 14.3 | 14.3 | 14.3 |  |
| Smoking | Yes | 19.6 | 19.6 | 24.6 | 17.9 | 18.3 | 0.32 | 15.8 | 20.4 | 21.3 | 21.3 | 21.3 | 0.01 |
|  | No | 23.3 | 21.9 | 17.8 | 24.0 | 13.0 |  | 23.3 | 24.0 | 20.5 | 19.9 | 12.3 |  |
| Education | None | 18.2 | 15.9 | 18.2 | 31.8 | 15.9 | 0.0004 | 15.9 | 13.6 | 20.5 | 29.5 | 20.5 | 0.002 |
|  | Vocational | 10.8 | 20.4 | 19.4 | 23.7 | 25.8 |  | 21.5 | 14.0 | 18.3 | 15.1 | 31.2 |  |
|  | Bachelor | 23.4 | 20.5 | 24.0 | 18.7 | 13.5 |  | 15.2 | 26.9 | 19.9 | 23.4 | 14.6 |  |
|  | Academic | 29.5 | 23.1 | 23.1 | 12.8 | 11.5 |  | 24.4 | 24.4 | 26.9 | 16.7 | 7.7 |  |
| Offspring’s physical activity | Sedentary - light | 25.4 | 15.3 | 25.4 | 18.6 | 15.3 | 0.96 | 27.1 | 15.3 | 18.6 | 25.4 | 13.6 | 0.73 |
|  | Light - moderate | 16.8 | 23.5 | 19.5 | 23.5 | 16.8 |  | 14.1 | 20.1 | 23.5 | 24.2 | 18.1 |  |
|  | Moderate - high | 30.6 | 14.5 | 27.4 | 14.5 | 12.9 |  | 17.7 | 30.6 | 16.1 | 21.0 | 14.5 |  |
|  | High | 19.0 | 22.4 | 20.7 | 19.8 | 18.1 |  | 20.7 | 22.4 | 21.6 | 13.8 | 21.6 |  |
| Sex | Male | 18.4 | 19.1 | 23.0 | 24.3 | 15.1 | 0.34 | 17.1 | 21.7 | 19.7 | 23.7 | 17.8 | 0.50 |
|  | Female | 22.6 | 21.4 | 21.4 | 17.5 | 17.1 |  | 19.7 | 21.8 | 21.8 | 18.8 | 17.9 |  |

^1^Associations between covariates in categories and dietary GI and GL are reported as P-trend from Mantel-Haentzel χ^2^-test for trend.

Table S2. MS markers in male and female offspring dependent on their mothers’ dietary GI in 2nd trimester. Shown are the mean differences in the outcome variables waist circumference and systolic and diastolic blood pressure (indicated by *) and mean ratio for all other log transformed outcome variables. The table includes figures from analyses of quintiles of GI, and from analyses of the data using GI as continuous variable (ratio or difference per 10U GI increment)^1^.

|  |  |  | |  | | Ratio or difference* (95% CI) | | | | |
| --- | --- | --- | --- | --- | --- | --- | --- | --- | --- | --- |
|  |  | GI quintile 1 | GI quintile 2 | | GI quintile 3 | | GI quintile 4 | GI quintile 5 | GI continuous | p-value |
|  | Mean ± SD | 60.8 + 3.6 | 67.4 + 1.3 | | 71.1 + 1.0 | | 74.5 + 1.1 | 80.0 + 2.7 | 70.5 ± 6.9 |  |
| Fasting glucose | Unadjusted | 1 | 0.99 (0.96, 1.02) | | 1.02 (0.99, 1.04) | | 1.03 (1.00, 1.05) | 1.00 (0.97, 1.03) | 1.01 (0.99, 1.02) | 0.42 |
|  | Adjusted | 1 | 0.99 (0.97, 1.01) | | 1.01 (0.99, 1.04) | | 1.03 (1.00, 1.05) | 1.00 (0.98, 1.03) | 1.01 (1.00, 1.02) | 0.18 |
| Triglycerides | Unadjusted | 1 | 1.04 (0.91, 1.18) | | 1.10 (0.97, 1.24) | | 1.12 (0.98, 1.27) | 1.02 (0.89, 1.17) | 1.03 (0.97, 1.10) | 0.34 |
|  | Adjusted | 1 | 1.04 (0.91, 1.18) | | 1.10 (0.97, 1.24) | | 1.16 (1.02, 1.32) | 1.04 (0.90, 1.19) | 1.04 (0.98, 1.11) | 0.18 |
| HDL cholesterol | Unadjusted | 1 | 1.05 (0.98, 1.12) | | 1.03 (0.96, 1.10) | | 1.01 (0.95, 1.09) | 1.02 (0.95, 1.10) | 1.02 (0.99, 1.06) | 0.25 |
|  | Adjusted | 1 | 1.05 (0.98, 1.12) | | 1.04 (0.98, 1.11) | | 1.05 (0.98, 1.12) | 1.03 (0.96, 1.11) | 1.03 (1.00, 1.06) | 0.09 |
| LDL cholesterol | Unadjusted | 1 | 1.07 (0.97, 1.16) | | 1.06 (0.97, 1.16) | | 1.10 (1.01, 1.20) | 1.07 (0.97, 1.18) | 1.03 (0.98, 1.07) | 0.21 |
|  | Adjusted | 1 | 1.05 (0.96, 1.15) | | 1.07 (0.98, 1.16) | | 1.10 (1.01, 1.21) | 1.07 (0.97, 1.18) | 1.03 (0.98, 1.08) | 0.26 |
| Total cholesterol | Unadjusted | 1 | 1.05 (0.99, 1.11) | | 1.05 (0.99, 1.11) | | 1.07 (1.01, 1.13) | 1.05 (0.98, 1.11) | 1.03 (1.00, 1.06) | 0.10 |
|  | Adjusted | 1 | 1.04 (0.98, 1.10) | | 1.06 (1.00, 1.12) | | 1.09 (1.03, 1.15) | 1.05 (0.99, 1.12) | 1.03 (1.00, 1.06) | 0.05 |
| Systolic blood pressure * | Unadjusted | 0 | -2.93 (-6.19, 0.33) | | -0.73 (-3.93, 2.48) | | 1.07 (-2.20, 4.35) | -2.31 (-5.77, 1.16) | -0.55 (-2.17, 1.07) | 0.51 |
|  | Adjusted | 0 | -2.58 (-5.27, 0.10) | | -1.31 (-3.93, 1.30) | | -0.10 (-2.84, 2.63) | -2.11 (-5.02, 0.81) | -0.55 (-1.91, 0.82) | 0.43 |
| Diastolic blood pressure* | Unadjusted | 0 | -0.50 (-2.55, 1.54) | | 0.19 (-1.83, 2.20) | | 0.76 (-1.30, 2.81) | -0.37 (-2.55, 1.80) | 0.21 (-0.80, 1.22) | 0.69 |
|  | Adjusted | 0 | -0.21 (-2.25, 1.83) | | 0.33 (-1.66, 2.31) | | 1.15 (-0.92, 3.22) | -0.25 (-2.46, 1.95) | 0.29 (-0.74, 1.32) | 0.59 |
| Waist circumference* | Unadjusted | 0 | 0.27 (-1.20, 1.74) | | 0.60 (-0.85, 2.04) | | 1.48 (0.00, 2.95) | 1.43 (-0.13, 2.99) | 0.67 (-0.06, 1.39) | 0.07 |
|  | Adjusted | 0 | 0.28 (-1.08, 1.65) | | 0.32 (-1.01, 1.64) | | 0.71 (-0.68, 2.10) | 1.14 (-0.34, 2.62) | 0.47 (-0.22, 1.16) | 0.18 |
| BMI | Unadjusted | 1 | 0.98 (0.94, 1.02) | | 1.02 (0.98, 1.06) | | 1.03 (0.99, 1.07) | 1.00 (0.96, 1.05) | 1.01 (0.99, 1.03) | 0.44 |
|  | Adjusted | 1 | 0.99 (0.95, 1.03) | | 1.02 (0.98, 1.06) | | 1.03 (0.99, 1.08) | 1.01 (0.97, 1.05) | 1.01 (0.99, 1.03) | 0.28 |
| HOMA-IR | Unadjusted | 1 | 1.03 (0.89, 1.18) | | 1.17 (1.02, 1.33) | | 1.25 (1.09, 1.43) | 1.09 (0.95, 1.26) | 1.07 (1.00, 1.15) | 0.04 |
|  | Adjusted | 1 | 1.04 (0.91, 1.19) | | 1.16 (1.01, 1.32) | | 1.27 (1.11, 1.46) | 1.11 (0.96, 1.28) | 1.09 (1.01, 1.16) | 0.02 |
| Insulin | Unadjusted | 1 | 1.07 (0.94, 1.22) | | 1.14 (1.01, 1.30) | | 1.23 (1.08, 1.41) | 1.10 (0.96, 1.27) | 1.07 (1.01, 1.15) | 0.03 |
|  | Adjusted | 1 | 1.08 (0.94, 1.23) | | 1.15 (1.01, 1.30) | | 1.26 (1.11, 1.44) | 1.12 (0.97, 1.29) | 1.09 (1.02, 1.16) | 0.01 |
| Leptin | Unadjusted | 1 | 0.92 (0.63, 1.33) | | 1.12 (0.78, 1.62) | | 1.11 (0.76, 1.61) | 1.15 (0.77, 1.72) | 1.16 (0.96, 1.39) | 0.12 |
|  | Adjusted | 1 | 0.93 (0.72, 1.21) | | 1.22 (0.95, 1.57) | | 1.38 (1.06, 1.79) | 1.21 (0.91, 1.60) | 1.21 (1.06, 1.38) | 0.01 |

^1^Adjustment for potential confounding by multiple linear regression including energy intake, pre-pregnancy BMI (kg/m^2^), height (cm), smoking, education, and offspring sex and leisure activity. The p-value is the result of analyses of the data using GI as continuous variable (n=386). GI quintiles were determined from the original data files including 894 women.

Table S3. MS markers in female offspring dependent on their mothers’ dietary GI in 2nd trimester. Shown are the mean differences in the outcome variables waist circumference and systolic and diastolic blood pressure (indicated by *) and mean ratio for all other log transformed outcome variables. The table includes figures from analyses of quintiles of GI, and from analyses of the data using GI as continuous variable (ratio or difference per 10U GI increment)^1^.

|  |  |  | |  | | Ratio or difference* (95% CI) | | | | |
| --- | --- | --- | --- | --- | --- | --- | --- | --- | --- | --- |
|  |  | GI quintile 1 | GI quintile 2 | | GI quintile 3 | | GI quintile 4 | GI quintile 5 | GI continuous | p-value |
|  | Mean ± SD | 60.8 + 3.6 | 67.4 + 1.3 | | 71.1 + 1.0 | | 74.5 + 1.1 | 80.0 + 2.7 | 70.4 + 6.7 |  |
| Fasting glucose | Unadjusted | 1 | 0.98 (0.95, 1.01) | | 1.01 (0.98, 1.04) | | 1.02 (0.99, 1.05) | 0.99 (0.96, 1.02) | 1.00 (0.99, 1.02) | 0.88 |
|  | Adjusted | 1 | 0.98 (0.95, 1.02) | | 1.01 (0.98, 1.05) | | 1.02 (0.99, 1.06) | 0.99 (0.96, 1.03) | 1.00 (0.99, 1.02) | 0.76 |
| Triglycerides | Unadjusted | 1 | 1.09 (0.92, 1.28) | | 1.09 (0.93, 1.29) | | 1.17 (0.99, 1.39) | 1.03 (0.86, 1.22) | 1.03 (0.95, 1.12) | 0.47 |
|  | Adjusted | 1 | 1.07 (0.91, 1.26) | | 1.08 (0.92, 1.28) | | 1.21 (1.01, 1.45) | 1.02 (0.85, 1.22) | 1.04 (0.95, 1.13) | 0.37 |
| HDL cholesterol | Unadjusted | 1 | 1.05 (0.97, 1.13) | | 1.02 (0.94, 1.10) | | 1.05 (0.96, 1.14) | 1.04 (0.96, 1.13) | 1.03 (0.99, 1.08) | 0.11 |
|  | Adjusted | 1 | 1.05 (0.97, 1.14) | | 1.02 (0.94, 1.11) | | 1.07 (0.98, 1.17) | 1.05 (0.96, 1.15) | 1.04 (1.00, 1.09) | 0.06 |
| LDL cholesterol | Unadjusted | 1 | 1.10 (0.98, 1.23) | | 1.06 (0.95, 1.19) | | 1.14 (1.01, 1.29) | 1.17 (1.03, 1.32) | 1.06 (1.00, 1.13) | 0.05 |
|  | Adjusted | 1 | 1.08 (0.96, 1.21) | | 1.06 (0.94, 1.20) | | 1.14 (1.00, 1.29) | 1.15 (1.01, 1.31) | 1.05 (0.99, 1.12) | 0.10 |
| Total cholesterol | Unadjusted | 1 | 1.08 (1.00, 1.16) | | 1.05 (0.97, 1.13) | | 1.11 (1.03, 1.20) | 1.11 (1.03, 1.20) | 1.05 (1.01, 1.09) | 0.01 |
|  | Adjusted | 1 | 1.06 (0.99, 1.15) | | 1.05 (0.97, 1.13) | | 1.12 (1.03, 1.22) | 1.11 (1.02, 1.20) | 1.05 (1.01, 1.09) | 0.01 |
| Systolic blood pressure* | Unadjusted | 0 | -2.53 (-5.73, 0.66) | | -1.81 (-5.01, 1.38) | | -0.23 (-3.60, 3.14) | -1.83 (-5.23, 1.57) | -0.61 (-2.22, 1.00) | 0.46 |
|  | Adjusted | 0 | -2.16 (-5.44, 1.11) | | -1.86 (-5.13, 1.42) | | -0.34 (-3.94, 3.25) | -1.50 (-5.11, 2.11) | -0.51 (-2.23, 1.21) | 0.56 |
| Diastolic blood pressure* | Unadjusted | 0 | 0.35 (-2.15, 2.84) | | 0.33 (-2.17, 2.82) | | 2.07 (-0.57, 4.70) | 0.81 (-1.84, 3.46) | 0.40 (-0.86, 1.66) | 0.53 |
|  | Adjusted | 0 | 0.58 (-1.93, 3.09) | | 0.11 (-2.40, 2.62) | | 2.07 (-0.68, 4.82) | 0.98 (-1.79, 3.74) | 0.52 (-0.80, 1.84) | 0.44 |
| Waist circumference* | Unadjusted | 0 | 1.07 (-0.75, 2.88) | | 1.18 (-0.64, 3.00) | | 1.73 (-0.19, 3.64) | 2.03 (0.10, 3.96) | 0.87 (-0.04, 1.78) | 0.06 |
|  | Adjusted | 0 | 1.03 (-0.78, 2.85) | | 1.04 (-0.78, 2.86) | | 1.18 (-0.82, 3.17) | 2.00 (-0.01, 4.00) | 0.78 (-0.17, 1.74) | 0.11 |
| BMI | Unadjusted | 1 | 0.99 (0.94, 1.05) | | 1.02 (0.96, 1.07) | | 1.03 (0.97, 1.09) | 1.01 (0.96, 1.07) | 1.00 (0.98, 1.03) | 0.79 |
|  | Adjusted | 1 | 1.00 (0.95, 1.05) | | 1.02 (0.96, 1.07) | | 1.04 (0.98, 1.10) | 1.02 (0.96, 1.08) | 1.01 (0.98, 1.04) | 0.52 |
| HOMA-IR | Unadjusted | 1 | 1.11 (0.93, 1.33) | | 1.24 (1.04, 1.47) | | 1.27 (1.06, 1.52) | 1.11 (0.92, 1.33) | 1.06 (0.97, 1.16) | 0.17 |
|  | Adjusted | 1 | 1.14 (0.95, 1.36) | | 1.23 (1.03, 1.46) | | 1.33 (1.10, 1.61) | 1.13 (0.93, 1.37) | 1.08 (0.99, 1.19) | 0.10 |
| Insulin | Unadjusted | 1 | 1.18 (1.00, 1.39) | | 1.23 (1.04, 1.45) | | 1.22 (1.03, 1.46) | 1.13 (0.95, 1.35) | 1.06 (0.97, 1.15) | 0.18 |
|  | Adjusted | 1 | 1.21 (1.03, 1.43) | | 1.22 (1.03, 1.44) | | 1.29 (1.08, 1.55) | 1.17 (0.97, 1.40) | 1.08 (0.99, 1.19) | 0.07 |
| Leptin | Unadjusted | 1 | 1.03 (0.78, 1.36) | | 1.21 (0.92, 1.60) | | 1.42 (1.06, 1.90) | 1.35 (1.00, 1.82) | 1.19 (1.03, 1.37) | 0.02 |
|  | Adjusted | 1 | 1.04 (0.79, 1.38) | | 1.19 (0.90, 1.58) | | 1.42 (1.05, 1.94) | 1.40 (1.03, 1.91) | 1.21 (1.05, 1.40) | 0.01 |

^1^ Adjustment for potential confounding by multiple linear regression including energy intake, pre-pregnancy BMI (kg/m^2^), height (cm), smoking, education, and offspring leisure activity. The p-value is the result of analyses of the data using GI as continuous variable (n=234). GI quintiles were determined from the original data files including 894 women.

Table S4. MS markers in male offspring dependent on their mothers’ dietary GI in 2nd trimester. Shown are the mean differences in the outcome variables waist circumference and systolic and diastolic blood pressure (indicated by *) and mean ratio for all other log transformed outcome variables. The table includes figures from analyses of quintiles of GI, and from analyses of the data using GI as continuous variable (ratio or difference per 10U GI increment)^1^.

|  |  |  | |  | | Ratio or difference* (95% CI) | | | | |
| --- | --- | --- | --- | --- | --- | --- | --- | --- | --- | --- |
|  |  | GI quintile 1 | Gi quintile 2 | | GI quintile 3 | | Gi quintile 4 | GI quintile 5 | GI continuous | p-value |
|  | Mean ± SD | 60.8 + 3.6 | 67.4 + 1.3 | | 71.1 + 1.0 | | 74.5 + 1.1 | 80.0 + 2.7 | 70.6 ± 6.4 |  |
| Fasting glucose | Unadjusted | 1 | 1.00 (0.96, 1.04) | | 1.02 (0.98, 1.06) | | 1.02 (0.98, 1.06) | 1.01 (0.97, 1.05) | 1.01 (0.99, 1.03) | 0.25 |
|  | Adjusted | 1 | 1.00 (0.96, 1.04) | | 1.02 (0.98, 1.06) | | 1.03 (0.99, 1.07) | 1.03 (0.98, 1.07) | 1.02 (1.00, 1.04) | 0.06 |
| Triglycerides | Unadjusted | 1 | 0.97 (0.80, 1.18) | | 1.12 (0.93, 1.35) | | 1.10 (0.92, 1.32) | 1.03 (0.84, 1.26) | 1.04 (0.95, 1.14) | 0.43 |
|  | Adjusted | 1 | 0.99 (0.80, 1.21) | | 1.14 (0.94, 1.38) | | 1.11 (0.92, 1.35) | 1.09 (0.87, 1.35) | 1.06 (0.96, 1.17) | 0.26 |
| HDL cholesterol | Unadjusted | 1 | 1.06 (0.95, 1.18) | | 1.07 (0.97, 1.19) | | 1.03 (0.93, 1.14) | 1.01 (0.90, 1.13) | 1.01 (0.96, 1.06) | 0.74 |
|  | Adjusted | 1 | 1.04 (0.93, 1.17) | | 1.07 (0.96, 1.19) | | 1.02 (0.92, 1.14) | 0.99 (0.88, 1.12) | 1.01 (0.95, 1.06) | 0.82 |
| LDL cholesterol | Unadjusted | 1 | 1.01 (0.88, 1.15) | | 1.06 (0.93, 1.21) | | 1.06 (0.93, 1.20) | 0.93 (0.81, 1.07) | 0.98 (0.92, 1.05) | 0.59 |
|  | Adjusted | 1 | 1.02 (0.88, 1.18) | | 1.08 (0.94, 1.24) | | 1.06 (0.93, 1.21) | 0.96 (0.83, 1.12) | 0.99 (0.93, 1.07) | 0.87 |
| Total cholesterol | Unadjusted | 1 | 1.01 (0.93, 1.10) | | 1.07 (0.98, 1.16) | | 1.04 (0.96, 1.13) | 0.95 (0.87, 1.04) | 0.99 (0.95, 1.03) | 0.71 |
|  | Adjusted | 1 | 1.01 (0.92, 1.11) | | 1.08 (0.99, 1.17) | | 1.04 (0.96, 1.14) | 0.97 (0.88, 1.07) | 1.00 (0.95, 1.04) | 0.96 |
| Systolic blood pressure* | Unadjusted | 0 | -4.37 (-9.24, 0.50) | | -1.26 (-5.92, 3.40) | | -1.00 (-5.60, 3.61) | -3.82 (-9.00, 1.35) | -0.91 (-3.23, 1.41) | 0.44 |
|  | Adjusted | 0 | -3.59 (-8.52, 1.33) | | -1.92 (-6.50, 2.66) | | -1.01 (-5.63, 3.61) | -4.00 (-9.23, 1.23) | -1.14 (-3.50, 1.21) | 0.34 |
| Diastolic blood pressure* | Unadjusted | 0 | -1.94 (-5.42, 1.54) | | 0.08 (-3.25, 3.41) | | -0.53 (-3.82, 2.76) | -2.40 (-6.09, 1.30) | -0.02 (-1.68, 1.63) | 0.98 |
|  | Adjusted | 0 | -1.28 (-4.87, 2.30) | | -0.13 (-3.47, 3.21) | | -0.94 (-4.31, 2.43) | -2.26 (-6.08, 1.55) | -0.21 (-1.92, 1.51) | 0.81 |
| Waist circumference* | Unadjusted | 0 | -1.41 (-3.50, 0.68) | | -1.07 (-3.07, 0.93) | | -0.21 (-2.19, 1.76) | 0.11 (-2.11, 2.34) | 0.22 (-0.78, 1.21) | 0.67 |
|  | Adjusted | 0 | -1.55 (-3.73, 0.63) | | -1.20 (-3.22, 0.83) | | -0.52 (-2.56, 1.52) | -0.81 (-3.12, 1.50) | -0.19 (-1.23, 0.85) | 0.72 |
| BMI | Unadjusted | 1 | 0.96 (0.90, 1.03) | | 1.02 (0.96, 1.08) | | 1.02 (0.96, 1.08) | 0.99 (0.92, 1.06) | 1.01 (0.98, 1.04) | 0.38 |
|  | Adjusted | 1 | 0.98 (0.92, 1.04) | | 1.02 (0.96, 1.08) | | 1.02 (0.96, 1.08) | 1.00 (0.93, 1.07) | 1.02 (0.99, 1.05) | 0.32 |
| HOMA-IR | Unadjusted | 1 | 0.89 (0.72, 1.11) | | 1.06 (0.85, 1.30) | | 1.21 (0.98, 1.50) | 1.06 (0.84, 1.33) | 1.09 (0.99, 1.21) | 0.09 |
|  | Adjusted | 1 | 0.89 (0.71, 1.11) | | 1.06 (0.86, 1.30) | | 1.23 (1.00, 1.52) | 1.05 (0.83, 1.32) | 1.09 (0.98, 1.22) | 0.10 |
| Insulin | Unadjusted | 1 | 0.90 (0.73, 1.12) | | 1.04 (0.85, 1.28) | | 1.25 (1.02, 1.53) | 1.05 (0.84, 1.32) | 1.10 (0.99, 1.22) | 0.07 |
|  | Adjusted | 1 | 0.88 (0.71, 1.10) | | 1.04 (0.85, 1.27) | | 1.24 (1.01, 1.52) | 1.01 (0.80, 1.27) | 1.08 (0.97, 1.20) | 0.14 |
| Leptin | Unadjusted | 1 | 0.83 (0.49, 1.38) | | 1.29 (0.78, 2.12) | | 1.33 (0.82, 2.18) | 1.02 (0.59, 1.77) | 1.22 (0.95, 1.56) | 0.11 |
|  | Adjusted | 1 | 0.78 (0.46, 1.31) | | 1.20 (0.73, 1.96) | | 1.20 (0.74, 1.97) | 0.91 (0.52, 1.59) | 1.14 (0.88, 1.46) | 0.31 |

^1^ Adjustment for potential confounding by multiple linear regression including energy intake, pre-pregnancy BMI (kg/m^2^), height (cm), smoking, education, and offspring leisure activity. The p-value is the result of analyses of the data using GI as continuous variable (n=152). GI quintiles were determined from the original data files including 894 women.
