# Supplementary material for: Dietary Glycemic Index during Pregnancy Is Associated with Biomarkers of the Metabolic Syndrome in Offspring at Age 20 Years
Source: PLoS One. 2013 May 31;8(5):e64887. doi: 10.1371/journal.pone.0064887 (PMC3669023; doi:10.1371/journal.pone.0064887)
Supplement: Table S2 — MS markers in male and female offspring dependent on their mothers’ dietary GI in 2nd trimester. (DOCX) [file pone.0064887.s002.docx]

ONLINE SUPPLEMENTARY DATA

Table S2. MS markers in male and female offspring dependent on their mothers’ dietary GI in 2nd trimester. Shown are the mean differences in the outcome variables waist circumference and systolic and diastolic blood pressure (indicated by *) and mean ratio for all other log transformed outcome variables. The table includes figures from analyses of quintiles of GI, and from analyses of the data using GI as continuous variable (ratio or difference per 10U GI increment)^1^.

|  |  |  | |  | | Ratio or difference* (95% CI) | | | | |
| --- | --- | --- | --- | --- | --- | --- | --- | --- | --- | --- |
|  |  | GI quintile 1 | GI quintile 2 | | GI quintile 3 | | GI quintile 4 | GI quintile 5 | GI continuous | p-value |
|  | Mean ± SD | 60.8 + 3.6 | 67.4 + 1.3 | | 71.1 + 1.0 | | 74.5 + 1.1 | 80.0 + 2.7 | 70.5 ± 6.9 |  |
| Fasting glucose | Unadjusted | 1 | 0.99 (0.96, 1.02) | | 1.02 (0.99, 1.04) | | 1.03 (1.00, 1.05) | 1.00 (0.97, 1.03) | 1.01 (0.99, 1.02) | 0.42 |
|  | Adjusted | 1 | 0.99 (0.97, 1.01) | | 1.01 (0.99, 1.04) | | 1.03 (1.00, 1.05) | 1.00 (0.98, 1.03) | 1.01 (1.00, 1.02) | 0.18 |
| Triglycerides | Unadjusted | 1 | 1.04 (0.91, 1.18) | | 1.10 (0.97, 1.24) | | 1.12 (0.98, 1.27) | 1.02 (0.89, 1.17) | 1.03 (0.97, 1.10) | 0.34 |
|  | Adjusted | 1 | 1.04 (0.91, 1.18) | | 1.10 (0.97, 1.24) | | 1.16 (1.02, 1.32) | 1.04 (0.90, 1.19) | 1.04 (0.98, 1.11) | 0.18 |
| HDL cholesterol | Unadjusted | 1 | 1.05 (0.98, 1.12) | | 1.03 (0.96, 1.10) | | 1.01 (0.95, 1.09) | 1.02 (0.95, 1.10) | 1.02 (0.99, 1.06) | 0.25 |
|  | Adjusted | 1 | 1.05 (0.98, 1.12) | | 1.04 (0.98, 1.11) | | 1.05 (0.98, 1.12) | 1.03 (0.96, 1.11) | 1.03 (1.00, 1.06) | 0.09 |
| LDL cholesterol | Unadjusted | 1 | 1.07 (0.97, 1.16) | | 1.06 (0.97, 1.16) | | 1.10 (1.01, 1.20) | 1.07 (0.97, 1.18) | 1.03 (0.98, 1.07) | 0.21 |
|  | Adjusted | 1 | 1.05 (0.96, 1.15) | | 1.07 (0.98, 1.16) | | 1.10 (1.01, 1.21) | 1.07 (0.97, 1.18) | 1.03 (0.98, 1.08) | 0.26 |
| Total cholesterol | Unadjusted | 1 | 1.05 (0.99, 1.11) | | 1.05 (0.99, 1.11) | | 1.07 (1.01, 1.13) | 1.05 (0.98, 1.11) | 1.03 (1.00, 1.06) | 0.10 |
|  | Adjusted | 1 | 1.04 (0.98, 1.10) | | 1.06 (1.00, 1.12) | | 1.09 (1.03, 1.15) | 1.05 (0.99, 1.12) | 1.03 (1.00, 1.06) | 0.05 |
| Systolic blood pressure * | Unadjusted | 0 | -2.93 (-6.19, 0.33) | | -0.73 (-3.93, 2.48) | | 1.07 (-2.20, 4.35) | -2.31 (-5.77, 1.16) | -0.55 (-2.17, 1.07) | 0.51 |
|  | Adjusted | 0 | -2.58 (-5.27, 0.10) | | -1.31 (-3.93, 1.30) | | -0.10 (-2.84, 2.63) | -2.11 (-5.02, 0.81) | -0.55 (-1.91, 0.82) | 0.43 |
| Diastolic blood pressure* | Unadjusted | 0 | -0.50 (-2.55, 1.54) | | 0.19 (-1.83, 2.20) | | 0.76 (-1.30, 2.81) | -0.37 (-2.55, 1.80) | 0.21 (-0.80, 1.22) | 0.69 |
|  | Adjusted | 0 | -0.21 (-2.25, 1.83) | | 0.33 (-1.66, 2.31) | | 1.15 (-0.92, 3.22) | -0.25 (-2.46, 1.95) | 0.29 (-0.74, 1.32) | 0.59 |
| Waist circumference* | Unadjusted | 0 | 0.27 (-1.20, 1.74) | | 0.60 (-0.85, 2.04) | | 1.48 (0.00, 2.95) | 1.43 (-0.13, 2.99) | 0.67 (-0.06, 1.39) | 0.07 |
|  | Adjusted | 0 | 0.28 (-1.08, 1.65) | | 0.32 (-1.01, 1.64) | | 0.71 (-0.68, 2.10) | 1.14 (-0.34, 2.62) | 0.47 (-0.22, 1.16) | 0.18 |
| BMI | Unadjusted | 1 | 0.98 (0.94, 1.02) | | 1.02 (0.98, 1.06) | | 1.03 (0.99, 1.07) | 1.00 (0.96, 1.05) | 1.01 (0.99, 1.03) | 0.44 |
|  | Adjusted | 1 | 0.99 (0.95, 1.03) | | 1.02 (0.98, 1.06) | | 1.03 (0.99, 1.08) | 1.01 (0.97, 1.05) | 1.01 (0.99, 1.03) | 0.28 |
| HOMA-IR | Unadjusted | 1 | 1.03 (0.89, 1.18) | | 1.17 (1.02, 1.33) | | 1.25 (1.09, 1.43) | 1.09 (0.95, 1.26) | 1.07 (1.00, 1.15) | 0.04 |
|  | Adjusted | 1 | 1.04 (0.91, 1.19) | | 1.16 (1.01, 1.32) | | 1.27 (1.11, 1.46) | 1.11 (0.96, 1.28) | 1.09 (1.01, 1.16) | 0.02 |
| Insulin | Unadjusted | 1 | 1.07 (0.94, 1.22) | | 1.14 (1.01, 1.30) | | 1.23 (1.08, 1.41) | 1.10 (0.96, 1.27) | 1.07 (1.01, 1.15) | 0.03 |
|  | Adjusted | 1 | 1.08 (0.94, 1.23) | | 1.15 (1.01, 1.30) | | 1.26 (1.11, 1.44) | 1.12 (0.97, 1.29) | 1.09 (1.02, 1.16) | 0.01 |
| Leptin | Unadjusted | 1 | 0.92 (0.63, 1.33) | | 1.12 (0.78, 1.62) | | 1.11 (0.76, 1.61) | 1.15 (0.77, 1.72) | 1.16 (0.96, 1.39) | 0.12 |
|  | Adjusted | 1 | 0.93 (0.72, 1.21) | | 1.22 (0.95, 1.57) | | 1.38 (1.06, 1.79) | 1.21 (0.91, 1.60) | 1.21 (1.06, 1.38) | 0.01 |

^1^Adjustment for potential confounding by multiple linear regression including energy intake, pre-pregnancy BMI (kg/m^2^), height (cm), smoking, education, and offspring sex and leisure activity. The p-value is the result of analyses of the data using GI as continuous variable (n=386). GI quintiles were determined from the original data files including 894 women.
